# Peripheral and Hepatic Vein Cytokine Levels in Correlation with Non-Alcoholic Fatty Liver Disease (NAFLD)-Related Metabolic, Histological, and Haemodynamic Features

**DOI:** 10.1371/journal.pone.0143380

**Published:** 2015-11-24

**Authors:** Luisa Vonghia, Thea Magrone, An Verrijken, Peter Michielsen, Luc Van Gaal, Emilio Jirillo, Sven Francque

**Affiliations:** 1 Department of Basic Medical Sciences, Neuroscience and Sensory Organs, University of Bari, Bari, Italy; 2 Department of Gastroenterology and Hepatology, University Hospital Antwerp, Antwerp, Belgium; 3 Department of Endocrinology, Diabetes and Metabolic Diseases, University Hospital Antwerp, Antwerp, Belgium; 4 Laboratory of Experimental Medicine and Paediatrics, Division of Gastroenterology, University of Antwerp, Antwerp, Belgium; Bambino Gesù Children's Hospital, ITALY

## Abstract

**Background:**

Haemodynamic impairment, inflammatory mediators and glucose metabolism disturbances have been implicated in the pathogenesis of Non-Alcoholic Fatty Liver Disease (NAFLD).

**Aim:**

To investigate the cytokine profile in NAFLD patients in peripheral (P) and hepatic venous (HV) blood and to compare with histology, haemodynamic and metabolic parameters.

**Methods:**

40 obese patients with an indication for a transjugular liver biopsy were enrolled. Besides an extended liver and metabolic work-up, interleukin (IL) 1B, IL4, IL6, IL10, IL23, tumour necrosis factor (TNF) α and interferon (INF) γ were measured in plasma obtained from P and HV blood by means of multiplex immunoassay. The T helper (Th)1/Th2, the macrophage M1/M2 and the IL10/IL17a ratios were calculated.

**Results:**

A decrease of the P-IL10/IL17-ratio and an increase of the P-M1/M2-ratio (p<0.05) were observed in NASH versus no-NASH patients. A P-M1/M2-ratio increase was detected also in patients with portal hypertension in comparison with patients without it (p<0.05). Moreover diabetic patients showed an increase of the P-Th1/Th2-ratio in comparison with non-diabetic ones (p<0.05). The P-M1/M2 ratio positively correlated with steatosis grade (r = 0.39, p = 0.02) and insulin (r = 0.47, p = 0.003). The HV-M1/M2 ratio positively correlated with fasting insulin and Hepatic Venous Pressure Gradient (r = 0.47, p = 0.003). IL6 correlated with the visceral fat amount (r = 0.36, p = 0.02). The P- and HV-IL10/IL17 ratios negatively correlated with fasting insulin (respectively r = -0.4, p = 0.005 and r = 0.4, p = 0.01).

**Conclusions:**

A proinflammatory cytokine state is associated with more disturbed metabolic, histological, and haemodynamic features in NAFLD obese patients. An increase of the M1/M2 ratio and a decrease of the IL10/IL17 ratio play a key role in this process.

## Introduction

Non-alcoholic fatty liver disease (NAFLD) is characterized by the presence of hepatic steatosis, in the absence of primary causes of hepatic fat accumulation. The estimated prevalence of NAFLD is 25–30% in Europe [1]. The severity of the disease can range from steatosis (Non-alcoholic fatty liver, NAFL) to Non-alcoholic Steatohepatitis (NASH), which can be accompanied by increasing grades of fibrosis. Five to 19% of NASH patients ultimately develop cirrhosis [2] that can lead to complications, including portal hypertension and hepatocellular carcinoma [3]. NAFLD is associated not only with liver-related morbidity and mortality, but also with an increased risk of developing cardiovascular disease, type 2 diabetes mellitus (DM) and the metabolic syndrome. Indeed, NAFLD is considered to be the hepatic component of the metabolic syndrome, as insulin resistance constitutes a common pathophysiological mechanism [4].

The pathogenesis of NASH is complex. According to the “multiple parallel hits hypothesis”, a number of different processes may contribute to liver injury [5]. Emerging evidence highlighted that increased portal pressure occurs in NAFLD, also in the absence of fibrosis, and that both dynamic factors with marked endothelial dysfunction and overproduction of vasoconstrictors, and morphological factors with pronounced architectural derangement of sinusoidal anatomy are implicated in its pathogenesis [6].

Furthermore a crucial role is played by the imbalance between pro-inflammatory and anti-inflammatory mechanisms, both from innate and adaptive immunity, which are involved in the induction and progression of liver and metabolic damage.

Macrophages display a heterogeneous behaviour, depending on the different environmental settings. Their activation ranges along a continuum between two separate polarization states: the “classically activated” pro-inflammatory M1 and the “alternatively activated” anti-inflammatory M2 states [7]. M1 polarized macrophages are induced by pro-inflammatory mediators, such as lipopolysaccharides (LPS) or interferon-γ (INFγ), and, in turn, lead to the secretion of pro-inflammatory cytokines, such as TNFα, IL6 and IL23 [8, 9]. M2 polarized macrophages can be induced by various stimuli, mostly IL4 and IL13 (but it can occur also in their absence), and produce anti-inflammatory cytokines as IL10 and IL-1 decoy receptor [10]. A stimulation of the pro-inflammatory M1 and a concordant decrease of the anti-inflammatory M2 have been reported in NASH and obesity [10, 11].

Depending on the cytokine environment, T helper lymphocytes can assume a pro-inflammatory phenotype (Th1), characterized by the release of INF-γ and transforming growth factor-β (TGF-β) or an anti-inflammatory phenotype (Th2), characterized by the release of IL4, IL5 and IL13. The equilibrium between Th1 and Th2 is important in driving the immune response. An imbalance between a relative excess of pro-inflammatory cytokines and a relative deficiency of anti-inflammatory cytokines has been found in the context of NASH both in the liver [12] and in the visceral adipose tissue [13]. Moreover Th1 enhancement can induce, via INF-γ, the infiltration of M1 polarized macrophages in obese mice [14].

The pro-inflammatory, IL17-producing effector T cells (Th17) are counterbalanced by the regulatory T cells (Tregs), which play a relevant role in the control of inflammation and can release IL10. Tregs are blunted by high fat diet in the setting of NASH [15], while an upregulation of the Th17 pathway occurs in fatty liver [16] and in liver fibrosis [17].

Much evidence shows that splanchnic inflammation contributes to the initiation and maintenance of portal hypertension, creating a loop between portal hypertension, splanchnic endothelial disorder, portal hypertensive enteropathy with inflammatory cell infiltration and gut microbiota alteration, systemic low-grade inflammation, and metabolic imbalance (due to a switch to predominant lipid metabolism) [18].

Cytokine imbalance is thus potentially implicated both in the pathogenesis of NASH and of portal hypertension. The liver acts as an “immunological organ”, giving rise to many processes implicated in inflammation and in immune control [19]. Nevertheless, the liver is not only an important site of synthesis but also the major clearance organ for several cytokines [20]. It has been demonstrated that hepatic uptake can influence the circulating levels of cytokines [21]. To our knowledge, no data are available regarding extensive cytokine evaluation in the different compartments (systemic versus intrahepatic) in NAFLD patients and their possible correlation with the liver-related and non-liver-related (metabolic and haemodynamic) components of the disease. The present study aims at giving insight into the cytokine profile in peripheral and hepatic venous blood of obese patients with an indication for a transjugular liver biopsy and to compare them with histology, haemodynamic and metabolic parameters.

## Patients and Methods

### Patients

Patients evaluated at the obesity clinic of the Antwerp University Hospital were prospectively recruited. They underwent a metabolic and hepatologic work-up. The study was approved by the Institutional Review Board of the University of Antwerp. All the patients signed a written informed consent [22, 23]. Exclusion criteria were significant alcohol consumption (self reported; > 20g/day), previous bariatric surgery, other chronic liver disease, already previously established diagnosis of diabetes, age < 16 years, pregnancy and absence of informed consent.

### Liver and metabolic assessment

Blood analysis included blood cell count, white blood cell formula, coagulation tests, thrombophilic tests, electrolytes and kidney function tests, liver enzyme tests [aspartate aminotransferase (AST), alanine aminotransferase (ALT), lactate dehydrogenase (LDH), gamma glutamyl transpeptidase (GGT), alkaline phosphatase (ALP), total bilirubin and fractions], creatinin kinase (CK), total protein, protein electrophoresis, thyroid function, sex hormones, ferritin, vitamin B and folic acid]. A 3 hour oral glucose tolerance test (OGTT) (75 g of glucose) including insulin and C-peptide analysis was performed and the haemoglobin A1C (HbA1C) was tested. Further examination included visceral fat measurement by computed tomography (CT) [24].

The liver specific assessment included additional blood analysis [s-choline-esterase, carcino-embryonic antigen, alpha-foetoprotein (AFP), anti-nuclear factor, anti-neutrophil cytoplasm antigen antibodies, anti-smooth muscle antibodies, anti-mitochondrial antibodies, anti liver-kidney microsomal antibodies, serum copper and ceruloplasmin, alpha-1-antitrypsin, HBsAg, anti HBcAb, anti Hepatitis C antibodies], a Doppler-ultrasound of the abdomen with parameters of liver and spleen size and liver vascularisation [25] and an aminopyrine breath test as a measure of hepatic metabolic reserve [26]. When one or more of the following criteria were met, raising the suspicion of NAFLD, a liver biopsy was proposed: abnormal liver enzyme tests [AST and/or ALT and/or GGT and/or (ALP)] and/or liver ultrasound abnormalities (steatotic liver) [27] and/or abnormal aminopyrine breath test [28]. For ALT 3 different cut-off levels were used: the upper limit of normal (ULN) set by the biochemistry laboratory (56 IU/L), the classical cut-off of 40 IU/L, and the limits proposed by Prati *et al*. (30 IU/L in men, 19 IU/L in women) [29]. Patients were proposed for transjugular liver venous catheterization and biopsy, as the transparietal route is technically less feasible in obese patients for anatomical reasons. Patients who were referred for bariatric surgery (including peri-operative liver biopsy) were excluded for further analysis.

### Transjugular liver biopsy

Transjugular liver vein catheterization and biopsy [30–33] were performed under fluoroscopic control and with permanent cardiovascular (mean arterial blood pressure, pulse rate and electrocardiogram) and respiratory monitoring (oxygen saturation, respiratory frequency). Free and wedged pressures were measured with a 5 French MP A2 Multipurpose Angiographic Catheter (Boston Scientific®, Nanterre, France). At least 3 measurements, each in a different branch of the right hepatic vein, were performed and averaged. Correct position of the wedged catheter and the presence/absence of collateral flow was checked by continuous fluoroscopic guidance and repetitive contrast injection to ascertain accuracy of the obtained wedged pressure values. Free pressures were measured in the main part of the right hepatic vein. The hepatic venous pressure gradient (HVPG) was calculated by subtracting the average free pressure from the average wedge pressure. A HVPG of > 5 mm Hg was used to define portal hypertension [31]. After the biopsy was performed using a 16 G Transjugular Liver Biopsy Needle (William Cook Europe®, Bjaeverskov, Denmark), a right heart catheterization was performed measuring central venous pressure (superior caval vein), right atrial pressure, right ventricular pressure and pulmonary artery pressure, as well as the cardiac output. Cardiac index (CI), systemic vascular resistance (SVR) and pulmonary vascular resistance (PVR) were subsequently calculated [34].

The liver biopsy specimen was stored in formalin aldehyde. Haematoxylin-eosin stain, Sirius red (Fouchet) stain, Periodic Acid Schiff stain after diastase, reticulin stain (Gordon-Sweets) and Perl’s iron stain were routinely performed on all biopsies and subsequently analyzed by an experienced pathologists, using the NASH Clinical Research Network Scoring System [35]. Steatosis was graded as follows: <5% of liver parenchyma: 0; 5–33%: 1; >33–66%: 2; >66%: 3. Lobular inflammation was scored: no foci: 0; <2 foci per 200x field: 1; 2–4 foci per 200x field: 2; >4 foci per 200x field: 3. Ballooning was scored: none: 0; few balloon cells: 1; many cells/prominent ballooning: 2. Fibrosis was staged: none: 0; perisinusoidal or periportal: 1; perisinusoidal and portal/periportal: 2; bridging fibrosis: 3; cirrhosis: 4. Other features (e.g. portal inflammation, Mallory’s hyaline) were even so assessed [35]. The NAFLD Activity Score (NAS) was calculated as the sum of the scores for steatosis, ballooning and lobular inflammation [35]. NASH was diagnosed if some degree of statosis, lobular inflammation and ballooning were simultaneously present [3, 36, 37].

### Cytokine evaluation

During the transjugular vein catheterization, before pressure measurement and biopsy, peripheral (P) blood samples and blood samples from the hepatic veins (HV) were collected. The cytokine analysis was performed with the Bioplex technology (BioRad Laboratories) combining the principle of a sandwich immunoassay with fluorescent bead-based technology, thus allowing individual and multiplex analysis of up to 100 different analytes in a single microtiter well [38]. The Bioplex assay for nine cytokines (IL1β, IFNγ, TNFα, IL4, IL6, IL10, IL17a, IL21 and IL23) was carried out in 96-well microplates using the 9-plex Bioplex Pro Human Th17 Cytokine Assay kit (Code YN00000SWY, Bio-Rad Laboratories) at the Bioclarma—Research and Molecular Diagnostics, Torino, Italy. Forty plasma samples from peripheral blood and 40 plasma samples from hepatic vein blood were diluted at 1:4 before the analysis and treated according to the manufacturer's instructions. The contents of each well were drawn up into the Bio-Plex 200 System array reader (Bio-Rad Laboratories), which identifies and quantifies each specific reaction based on bead colour and fluorescent signal intensity. The data were finally processed using Bio-Plex Manager software (version 6.1) using five-parametric curve fitting and converted in pg/mL. Delta (Δ) values were calculated as the ratio HV value/P value. To study the balance between pro- and anti-inflammatory systems the INFγ/IL4 ratio (as representative of the Th1/Th2 balance), the (TNFα+IL6+IL23)/IL10 ratio (as representative of the M1/M2 balance) and the IL10/IL17a ratio were calculated [39–41].

### Statistical analysis

Data are presented as mean ± standard deviation. Statistical differences were analyzed by student T test for independent variables (if normally distributed), Mann-Withney U test for independent variables (if not normally distributed) and paired T test or paired non-parametric test for paired data as appropriate; a value of p<0.05 was considered statistically significant. Correlations were calculated with the Spearman’s rank correlation coefficient. SPSS 20.0 software was used for all the statistic calculations.

## Results

### Metabolic and liver workup

Forty patients (18 men, 22 women; all Caucasian; mean age 47 years) were enrolled in the study. Table 1 summarizes the main characteristics of the patients. Patients were obese and had slightly disturbed liver tests, hypercholesterolaemia and hypertriglyceridaemia. Thirty % of the patients reached values at OGTT diagnostic for diabetes. Twenty % of the patients showed a HPVG compatible with portal hypertension, of which only one patient had a HVPG > 10 mmHg, compatible with clinically significant portal hypertension [42]. NASH was histologically diagnosed in 70% of the patients (Table 2). No patients had liver cirrhosis. The right heart catheterization showed high mean CVP values and high mean systemic vascular resistance (SVRI) in the overall patient group, and no pulmonary hypertension (Table 2). There were no differences in haemodynamic parameters measured during the right heart catheterization between patients without and with NASH and without and with portal hypertension.

**Table 1:** patients’ main characteristics.

		SD	Normal Values (range)
Sex (M/F)	18/22		
Age (years)	47	11	
Weight (kg)	116.15	20.95	
BMI (kg/m^2^)	39.78	6.64	<25
WHR	0.99	0.07	0.67–0.80
CRP (mg/dL)	0.84	0.90	<0.3
AST (IU/L)	40.85	26.01	<31
ALT (IU/L)	57.83	29.45	<34
GGT (IU/L)	46.88	32.21	12–38
Total bilirubin (mg/dL)	0.67	0.242	0.3–1.2
Triglycerides (mg/dL)	166.49	76.47	<150
Cholesterol (mg/dL)	204.25	47.11	150–220
HDL (mg/dL)	43.08	10.16	<40
LDL (mg/dL)	130.34	47.54	<115
Fasting glucose (md/dL)	87.48	11.17	74–100
Fasting insulin (mU/L)	18.89	8.924	2.6–24.9
Fasting C-peptide (nmol/L)	1.27	0.32	0.37–1.47
Haemoglobin A1C (%)	5.76	0.52	4.8–6
Blood pressure-systolic (mmHg)	132.54	14.52	100–139
Blood pressure-diastolic (mmHg)	78.15	11.41	70–89
Creatinine (mg/dL)	0.91	0.19	0.45–0.75
HOMA-IR	4.05	2.27	<2.5
HVPG	4.43	2.490	<5 mmHg
MAP	100.47	18.329	70–105 mmHg
CVP	9.67	4.182	2–6 mmHg
mPAP	21.21	8.298	10–20 mmHg
PCWP	14.05	6.1	6–12 mmHg
CI	2.6226	0.50	2.5–4 L/min/m^2^
SVRI	2895.48	670.66	1900–2400 dyn*s/ cm^5^*m^2^
Diabetes	12/40 (30%)		
NASH	28/40 (70%)		
Portal Hypertension	8/40 (20%)		

Number (n) or mean value ± standard deviation (SD). BMI: body mass index, AST: aspartate aminotransferase, ALT: alanine aminotransferase, WHR: waist-hip ratio, CRP: C-reactive protein, GGT: gamma glutamyl transpeptidase, HDL: high-density lipoprotein, LDL: low-density lipoprotein, HbA1C: haemoglobin A1C, HOMA-IR: homeostasis model assessment of insulin resistance, NASH: Non-alcoholic Steatohepatitis, HVPG: hepatic venous pressure gradient, MAP: mean arterial pressure, CVP: central venous pressure, mPAP: mean pulmonary artery pressure, PCWP: pulmonary capillary wedge pressure, CI: cardiac index, SVRI: systemic vascular resistance index.

**Table 2:** Liver histology.

Steatosis	
0	7/40 (17.5%)
1	12/40 (30%)
2	11/40 (27.5%)
3	10/40 (25%)
Hepatocellular ballooning	
0	7/40 (17.5%)
1	15/40 (37.5%)
2	18/40 (45%)
Lobular inflammation	
0	9/40 (22.5%)
1	20/40 (50%)
2	8/40 (20%)
3	3/40 (7.5%)
Fibrosis	
0	33/40 (82.5%)
1	2/40 (5%)
2	0
3	5/40 (12.5%)
4	0
Sinusoidal dilation	4/40 (10%)
Microvescicular steatosis	3/40 (7.5%)
Inflammatory microgranulomas	2/40 (5%)
Inflammatory large lipogranulomas	0/40 (0%)
Portal inflammation	6/40 (15%)
Acidophilic bodies	1/40 (2.5%)
Pigmented macrophages	2/40 (5%)
Megamitochondria	0/40 (0%)
Mallory’s hyaline	3/40 (7.5%)
Glycogenated nuclei	22/40 (24.5%)
Iron deposition	3/40 (7.5%)
NAS	4 ±2.2
NASH (n)	28/40 (70%)

Non-alcoholic Fatty Liver Disease (NAFLD) Activity Score (NAS) was calculated as the sum of the subscores for steatosis, lobular inflammation and ballooning.

### Cytokines

When considering the whole group of patients, no difference between cytokine-measurements in P blood and HV blood reached statistical significance, except for IL6 (paired samples test). IL6 was higher in P than in HV blood (respectively 8.84±2.6 and 7.04±1.97 pg/mL; p = 0.001) (Table 3).

**Table 3:** Cytokine levels (pg/mL) in the overall patient group.

	P blood	HV blood
IL23	5.50±5.10	4.99±5.31
IL1β	0.65±0.25	0.66±0.25
IL4	3.13±4.33	4.56±5.26
IL6	8.84±2.60	7.04±1.97 *
IL10	7.24±4.29	6.82±3.68
IL17a	4.42±1.29	4.39±1.18
IL21	20.04±21.30	19.37±21.28
INFγ	9.24±6.51	9.40±5.69
TNFα	4.50±1.08	4.43±1.38

Hepatic venous blood (HV) and peripheral (P) blood. IL: interleukin, INFγ: interferon γ, TNFα: tumour necrosis factor α.

* HV statistically significant compared to P.

Patients with and without histologically proven NASH, with and without portal hypertension and with and without DM were then separately analyzed.

When considering the single cytokines, patients with NASH displayed lower values of P-IL21 in comparison with patients without it (respectively 14.94±14.46 vs. 33.02±30.01 pg/mL; p = 0.015) (Table 4). Patients with advanced fibrosis (F3-F4) showed higher values of ΔINFγ and ΔIL1B (respectively 4.39±6.23 vs.1.35±1.19 pg/mL; p = 0.01 and 1.6±0.42 vs. 1.02±0.43 pg/mL; p = 0.01).

**Table 4:** Cytokine levels in different groups of patients.

	NO NASH	NASH	NO DM	DM	NO PH	PH
P-IL23	5.61±3.74	5.46±5.60	7.80±8.39	4.48±2.16	3.99±0.00	5.83±5.59
P-IL1β	0.77±0.35	0.61±0.19	0.75±0.34	0.61±0.19	0.5957±0.17	0.66±0.27
P-IL4	2.69±3.60	3.30±4.64	4.01±5.55	2.73±3.73	2.36±3.02	3.29±4.59
P-IL6	8.58±2.04	8.94±2.82	9.19±2.56	8.69±2.65	9.1257±3.97	8.78±2.29
P-IL10	9.27±4.50	6.45±4.00	8.43±4.59	6.72±4.12	6.75±3.32	7.35±4.51
P-IL17a	4.80±1.55	4.27±1.18	4.86±1.57	4.23±1.13	3.77±1.13	4.56±1.30
P-IL21	33.02±30.01	14.94±14.46 *	27.87±30.44	16.56±15.17	19.92±23.35	20.07±21.22
P-INFγ	10.80±7.65	8.62±6.05	11.05±7.47	8.43±6.02	7.09±3.84	9.71±6.92
P-TNFα	4.30±0.74	4.58±1.19	4.83±1.15	4.36±1.03	4.51±1.30	4.50±1.05
HV-IL23	6.43±9.35	4.33±1.36	3.93±1.10	5.42±6.25	7.99±11.32	4.19±1.39
HV-IL1β	0.67±0.25	0.65±0.25	0.77±0.22	0.61±0.25	0.59±0.22	0.68±0.25
HV-IL4	4.48±6.15	4.60±4.92	8.61±5.61 #	2.91±4.17	4.76±5.83	4.51±5.20
HV-IL6	6.89±1.97	7.11±2.01	7.71±2.10	6.76±1.89	7.42±1.86	6.94±2.02
HV-IL10	8.54±4.28	6.02±3.15	7.04±3.26	6.72±3.89	5.45±4.69	7.18±3.36
HV-IL17a	4.24±1.00	4.46±1.27	4.72±1.06	4.26±1.22	4.22±0.82	4.44±1.27
HV-IL21	20.28±18.14	18.95±22.92	24.60±26.26	17.24±19.06	12.70±11.65	21.15±23.01
HV-INFγ	9.68±7.05	9.27±5.10	12.03±4.00 §	8.33±5.98	7.68±3.21	9.86±6.14
HV-TNFα	4.59±1.95	4.36±1.06	4.53±1.02	4.39±1.52	4.29±0.64	4.47±1.52

Cytokine levels (pg/mL) in hepatic venous (HV) and peripheral (P) blood in patients respectively without an established diagnosis of Non-alcoholic Steatohepatitis (NASH) in comparison with NASH patients, patients without and with diabetes mellitus (DM) and patients without and with portal hypertension (PH). IL: interleukin, INFγ: interferonγ, TNFα: tumor necrosis factor α

*p = 0.015

#p = 0.008

§p = 0.03

The patients with DM showed higher levels of HV-IL4 and HV-INFγ than non-diabetic patients (respectively 8.61±5.61 vs. 2.91±4.17 pg/mL p = 0.008 and 12.03±4.00 vs. 8.33±5.98 pg/mL; p = 0.03) (Table 4). Moreover diabetic patients showed lower values of ΔIL23 and higher values of ΔIL4 than the non-diabetic ones (respectively 0.77±0.37 vs. 1.00±0.20 pg/mL; p = 0.02 and 7.25±7.66 vs. 2.04±4.07 pg/mL; p = 0.01) (Table 5).

**Table 5:** Δ cytokine levels in different groups of patients.

	NO NASH	NASH	NO DM	DM	NO PH	PH
Δ-IL23	0.85±0.32	0.96±0.26	1.00±0.20	0.77±0.37 #	0.91±0.31	1.00±0.00
Δ-IL1β	0.98±0.47	1.13±0.46	1.06±0.42	1.15±0.56	1.10±0.47	1.03±0.45
Δ-IL4	2.30±3.65	4.13±6.49	2.04±4.07	7.25±7.66 *	3.68±5.92	3.20±5.68
Δ-IL6	1.00±0.62	1.42±1.64	1.43±1.64	0.96±0.58	1.39±1.51	0.86±0.88
Δ-IL10	0.95±0.28	1.09±0.34	1.05±0.30	1.04±0.39	1.01±0.32	1.21±0.31
Δ-IL17a	0.80±0.27	0.87±0.39	0.83±0.35	0.89±0.37	0.85±0.37	0.84±0.27
Δ-IL21	1.40±2.28	1.56±1.80	1.52±2.02	1.51±1.77	1.66±2.12	0.90±0.25
Δ-INFγ	2.20±3.89	1.46±1.25	1.73±2.69	1.55±1.15	1.75±2.56	1.38±0.75
Δ-TNFα	1.07±0.50	0.95±0.13	1.00±0.33	0.94±0.15	0.99±0.32	0.95±0.13

Δ cytokine values, calculated as Hepatic venous value (pg/mL) /Peripheral value (pg/mL), in patients respectively without an established diagnosis of Non-alcoholic Steatohepatitis (NASH) in comparison with NASH patients, patients without and with diabetes mellitus (DM) and patients without and with portal hypertension (PH). IL: interleukin, INFγ: interferonγ, TNFα: tumour necrosis factor α.

# p = 0.02

* p = 0.01

Patients with DM and NASH had higher values of ΔIL4 than the no-NASH patients without diabetes (8.36±8.31 vs. 2.27±4.21 pg/mL; p = 0.007) (data not shown).

**Fig 1:**
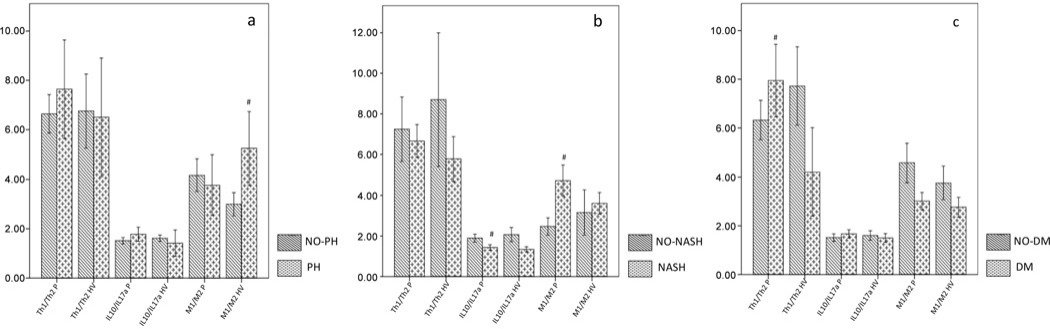
Th1/Th2, M1/M2 and IL10/IL17a ratios. Th1/Th2, M1/M2 and IL10/IL17a ratios in patients respectively without and with portal hypertension (PH) (a: NO PH *vs* PH), in patients without an established diagnosis of Non-alcoholic Steatohepatitis (NASH), in comparison with NASH patients (b: NO NASH *vs* NASH) and in patients without and with diabetes mellitus (DM) (c: NO DM *vs* DM). IL: interleukin, INFγ: interferonγ, TNFα: tumour necrosis factor α, #: statistically significant difference between groups (p<0.05).

**Fig 2:**
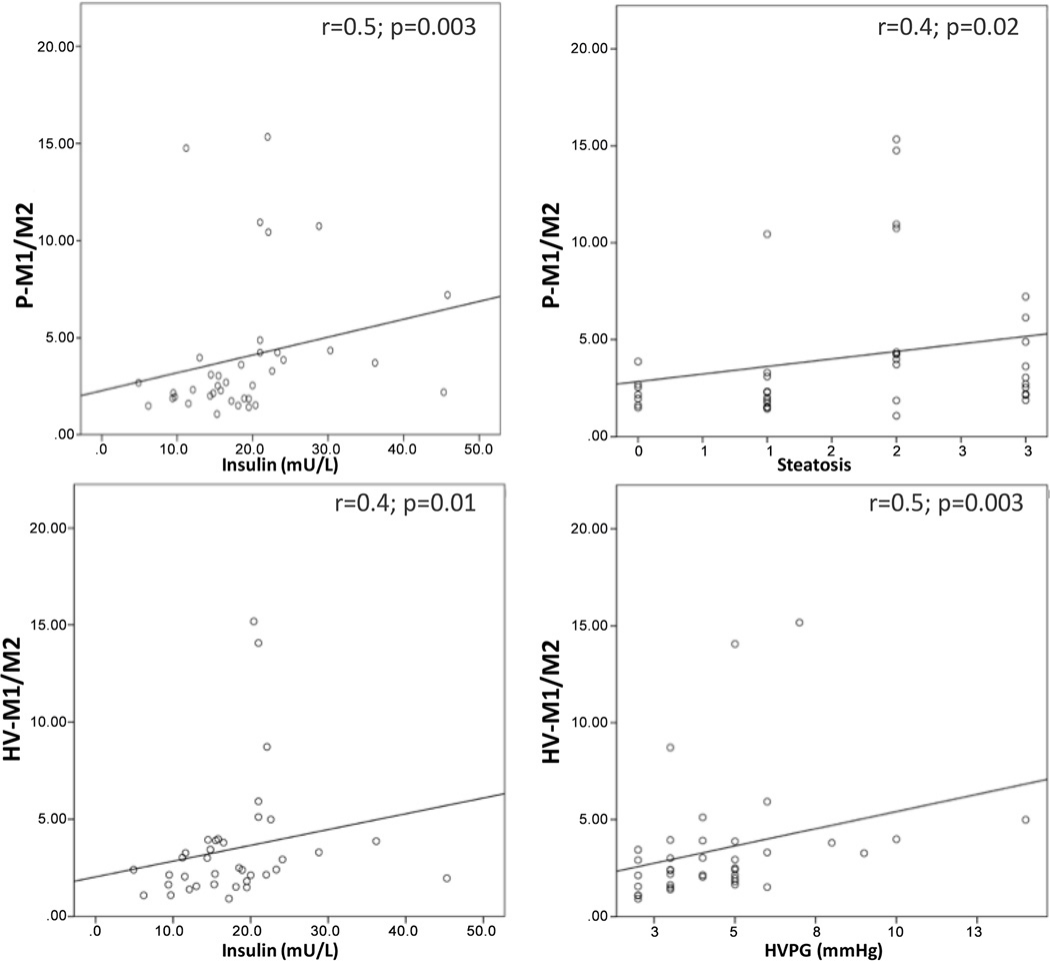
Significant correlations between M1/M2 ratio and clinical features. M1/M2 significant correlations with the respective correlation coefficient (p<0.05). The peripheral (P)-M1/M2 ratio positively correlated with steatosis grade and with fasting insulin. The hepatic venous (HV)-M1/M2 ratio positively correlated with fasting insulin.

When considering the ratios, a significantly lower P-IL10/IL17a-ratio (1.43±0.73 vs.1.89±0.64 pg/mL; p = 0.04) and higher P-M1/M2-ratio (3.86±2.72 vs. 2.21±1.11 pg/mL; p = 0.01) was observed in NASH versus no-NASH patients (Fig 1). The HV-M1/M2-ratio was also significantly lower in patients without portal hypertension in comparison with patients with this feature (2.99±2.57 vs. 5.24±4.21; p = 0.01) and in the overall group of patients the HV-M1/M2-ratio correlated positively with the HVPG (r = 0.47; p = 0.003) (Fig 2). Moreover diabetic patients showed an increase of the P-Th1/Th2-ratio in comparison with non-diabetic ones (7.94±5.15 vs. 6.32±4.2 pg/mL; p = 0.04) (Fig 1).

**Fig 3:**
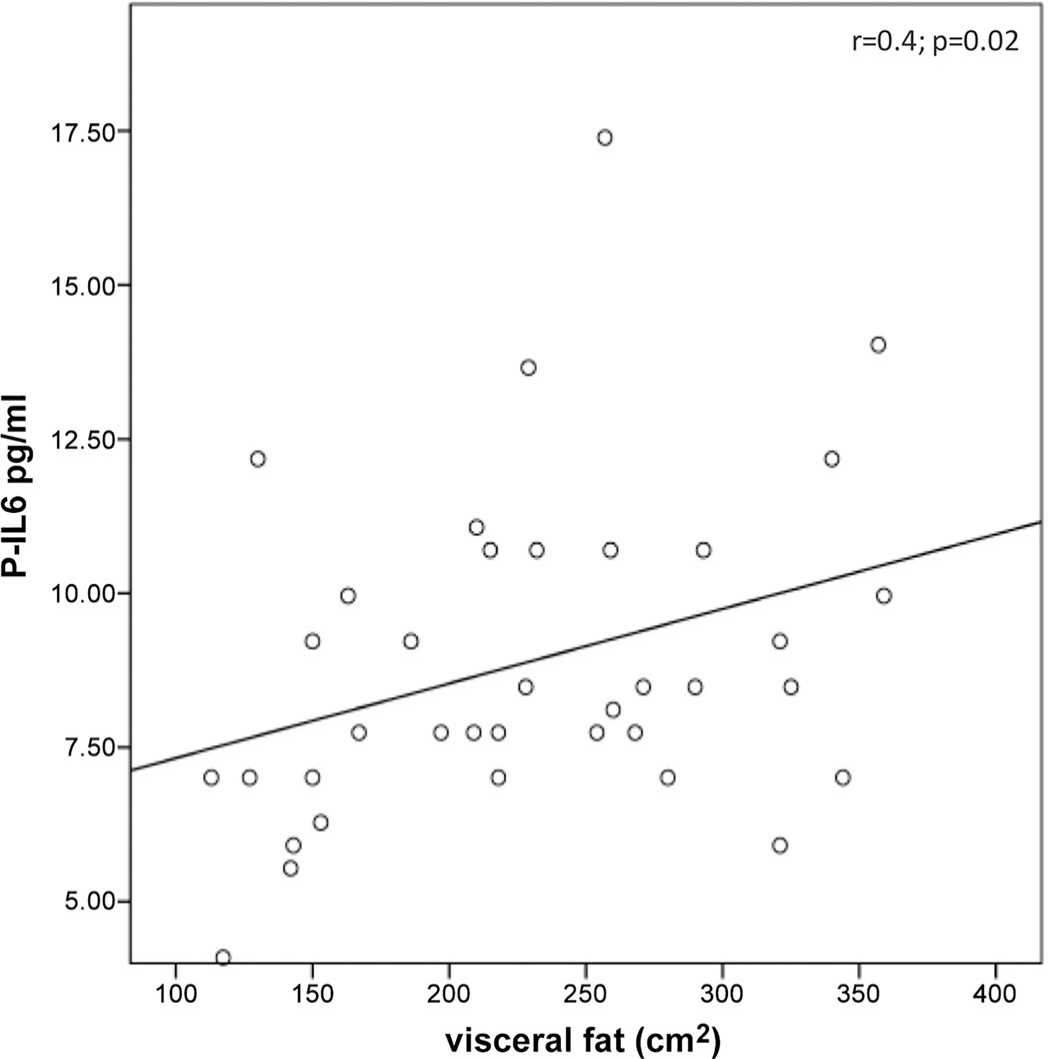
Significant correlations between IL6 levels and clinical features. IL6 level in peripheral blood (P) correlated with the visceral fat amount.

**Fig 4:**
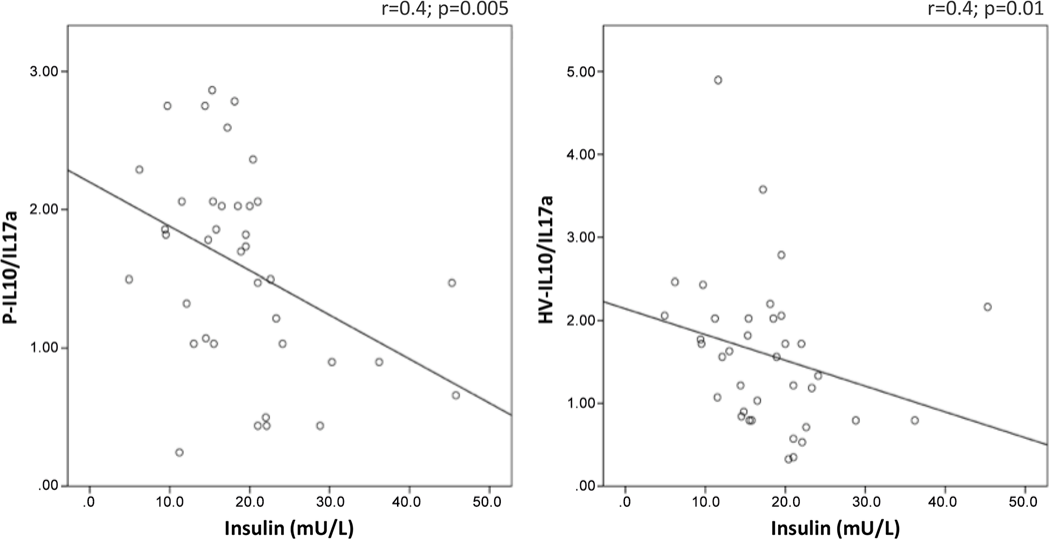
Significant correlations between IL10/IL17a ratio and clinical features. IL10/IL17a Significant correlation with the respective correlation coefficient (p<0.05). The peripheral (P)- and hepatic venous (HV)-IL10/IL17 ratios negatively correlated with fasting insulin.

The P-M1/M2 ratio positively correlated with steatosis grade (r = 0.39; p = 0.02) and with fasting insulin (r = 0.47; p = 0.003). The HV-M1/M2 ratio positively correlated with fasting insulin (r = 0.36; p = 0.02) (Fig 2). IL6 correlated with the visceral fat amount (r = 0.36; p = 0.02) (Fig 3). The P- and HV-IL10/IL17a ratios negatively correlated with fasting insulin (r = -0.4; p = 0.005 and r = -0.4; p = 0.01, respectively) (Fig 4). The average glucose levels in the last 120 days, represented by the HbA1C, positively correlated with both P- and HV-IL6 (respectively r = 0.36; p = 0.024 and r = 0.4; p = 0.01), with HV-IL1β (r = 0.36; p = 0.02), HV-IFNγ (r = 0.42; p = 0.008) and HV-TNFα (r = 0.41; p = 0.01).

Moreoever, when studying detailed features of liver histology and the cytokines, we observed a significant negative correlation between the sinusoidal dilatation and the P-IL10/IL17a ratio (r = -0.32; p = 0.04) and with P-IL10 (r = -0.38; p = 0.04). The presence of perisinusoidal fibrosis positively correlated with both P- and HV-TNFα (respectively r = 0.43; p = 0.01 and r = 0.39; p = 0.02). In addition, the presence of glycogenated nuclei positively correlated with the HV-IL6 (r = 0.35; p = 0.3). The NAS score and its subscores steatosis and ballooning as well as liver fibrosis inversely correlated with P-IL21 (respectively, r = -0.44; p = 0,005, r = -0.48; p = 0.002, r = -0.33; p = 0.04, r = -0.33; p = 0.03).

As to the biochemical liver tests, the transaminase levels (both AST and ALT) inversely correlated with P-Th1/Th2 (r = -0.41; p = 0.09, r = -0.35; p = 0.02), while bilirubin inversely correlated with HV-IL4 (r = -0.37; p = 0.02). Considering separately the NASH and NO-NASH patients, we found a positive correlation between P-IL10/IL17 and AST (r = 0.72; p = 0.01) in the NO-NASH patients and a positive correlation between HV-IL10/IL17 and AST (r = 0.48; p = 0.01) in the NASH patients. Moreover, there was a negative correlation between the ΔIL17 and AST (r = 0.45; p = 0.01) in the NASH patients.

Finally, when considering the haemodynamic values measured durning the right heart catheterization, we observed a postive correlation between MAP (mean arterial pressure) and P-IL4 (r = 0.39; p = 0.01) and with P-IL1B (r = 0.37; p = 0.03). CVP (central venous pressure) positively correlated with ∆IL1B (r = 0.40; p = 0.01) and the PCWP (pulmonary capillary wedge pressure) directly correlated with INFγ (r = 0.37; p = 0.02) and with ∆IL1B (r = 0.37; p = 0.02).

Linear regression showed that the ratio HV-IL10/IL17a was predictive of the presence of NASH (r^2^ = 0.14; p = 0.02).

## Discussion

There is a growing interest in the role of the immune system as a key contributor to the pathogenesis of NASH, metabolic syndrome and portal hypertension [7]. These different aspects are strictly linked together since NAFLD and hence NASH are currently considered as hepatic manifestations of the metabolic syndrome [43] and intrahepatic vascular alterations (manifested by the presence of some degree of portal hypertension) constitutes both a potential key player in the pathogenesis of NASH [6, 44, 45] and a consequence of advanced liver disease [46]. Increasing evidence highlights the role of a pro-inflammatory state in initiating and maintaining inflammation in the onset of NASH and its metabolic and haemodynamic consequences [7, 18].

In the present study we investigated the serum cytokine profile in obese patients with an indication to a transjugular liver biopsy in order to study the contribution of the immune mechanisms to the pathophysiology of NAFLD and hence NASH and to selective study liver-specific alterations by differentially examining both peripheral and hepatic venous blood. Peripheral blood is a mixture of venous blood coming from different organs and is hence not necessarily representative for what happens at the hepatic level. Hepatic venous blood represents the outflow tract of the liver and hence theoretically allows assessing more specifically liver site-specific mechanisms.

A more accentuated pro-inflammatory state (indicated by a decrease of the IL10/IL17a ratio) was found in NASH patients versus no-NASH patients. In agreement with our results, others have shown that Th17-related genes, including IL17, were upregulated in a high fat diet murine model, while IL17 neutralization attenuated LPS-induced liver injury [16]. In addition, the IL10/IL17a ratio negatively correlated with insulin levels, linking a pro-inflammatory state to increasing insulin resistance. Notably, both P- and HV-IL10/IL17a ratios showed this correlation. According to the linear regression analysis, HV-IL10/IL17a was predictive of the presence of NASH. It can hence be concluded that a disturbed IL10/IL17a balance, in favour of a pro-inflammatory state, is involved both in the pathophysiology of steatohepatitis and in the metabolic disturbances related to insulin resistance and that this is a liver-specific phenomenon.

It has been reported that anti-inflammatory mechanisms are involved in portal hypertension. An increase in Treg-frequency has been reported in patients with portal hypertension and hypersplenism, suggesting that they may take part in portal pressure regulation [47]. Our results, however, did not show an increase of the anti-inflammatory cytokines, such as IL10, in relation to portal hypertension.

Furthermore, the M1/M2 ratio showed a positive correlation with steatosis and a M1 prone status was found in NASH patients. These data are in line with diet-induced murine models of obesity, in which macrophages switch to the M1 profile, accumulate in the adipose tissue and are able to express pro-inflammatory genes [11]. Accordingly, in our study DM patients displayed relatively higher values of P-IL23 than HV-IL23 (as highlighted by a reduced ΔIL23 in comparison with non diabetic patients), suggesting a more relevant role of the systemic M1 profile in this setting. Moreover, IL6, and hence the M1 profile, correlated with visceral fat accumulation and with glucose metabolism impairment at different levels. HbA1C values, which reflect the long-term alterations in blood glucose levels, correlated both with P- and HV-IL6 and at liver histology, the glycogenated nuclei positively correlated with the HV-IL6. Moreover, besides the correlation with HV-IL6, the HbA1C values correlated with a pro-inflammatory intrahepatic cytokine environment, as shown by the correlation with HV-IL1β, HV-IFNγ and HV-TNFα,.

Intriguingly, in the present study a M1 profile was found also in patients with portal hypertension and the HV-M1/M2 ratio correlated with the HVPG. Macrophage activation has been reported to be correlated to HVPG and variceal bleeding in cirrhotic patients [48]. To our knowledge no previous data are reported on the possible association between a shift to M1 profile and portal hypertension.

In addition both the P- and HV-M1/M2 ratio positively correlated with the insulin levels, therefore M1 polarization was associated also to insulin resistance. Taken together these results suggest that M1 polarization plays a determinant role in NASH patients, not only in driving liver injury but also in the metabolic and haemodynamic components of the disease.

Moreover, patients with advanced fibrosis (F3-F4) showed higher intrahepatic INFγ and IL1B (as demonstrated by higher ΔINFγ and ΔIL1B). IL1B can stimulate the cells towards a pro-inflammatory Th1 phenotype, inducing also IFNγ secretion [49]. This mechanism could be involved in fibrogenesis in the advanced phases of NASH.

Besides to the enhanced M1 profile in patients with portal hypertension, microcirculatory disturbances (evidenced by the presence of sinusoidal dilatation), which have been reported to be associated to portal hypertension [44], negatively correlated with the P-IL10/IL17a ratio, hence with a more pronounced IL17. Moreover, the presence of perisinusoidal fibrosis positively correlated with the pro-inflammatory TNFα both in the peripheral and in the intrahepatic blood. Of note, no patients with liver cirrhosis where included in our series, therefore these findigs reinforce the link between the inflammatory and vascular damage in NAFLD, independently from the presence of cirrhosis. These results give further insight into the role of early microvascular alterations in NAFLD. Liver steatosis/steatohepatitis can induce, in the absence of fibrosis, a haemodynamically significant increase in intrahepatic resistance due to both altered microvascular architecture and functional factors, such as blunted response to insulin dependent vasodilation, vasoconstrictors overproduction and endothelial dysfunction, the latter being involved at a very early stage of liver damage, even before the development of inflammation [44, 50, 51]. Morover, human studies showed that steatohepatitis can induce significant portal hypertension together with splanchnic vasodilation and hyperdynamic circulation, in the absence of fibrosis [6]. Our results hence further add to the potential link between these haemodynamic alterations and inflammation in the onset of NAFLD.

Furthermore IL1B was associated with the haemodynamic values measured during the right heart catheterization. Interstingly, higher CVP and PWCP correlated with higher intrahepatic IL1B values (as shown by the postitive correlation with the ∆IL1B), indicating a potential link between the cardiovascular disease associated to NAFLD [52] and the liver-specific inflammation.

Finally, diabetic patients showed a disturbed P-Th1/Th2 balance towards Th1 polarization. When considering HV cytokine levels, both the HV-IL4 and HV-INFγ were higher in the DM than in the group without DM. Moreover, the ratio HV/P-IL4 (ΔIL4) was increased in patients with DM and in patients with DM and NASH. Our results hence showed an enhancement of the systemic pro-inflammatory Th1 state in DM. At the intrahepatic level, however, a mixed Th1 and Th2 impairment occurred (as demonstrated by the higher HV-INFγ and HV-IL4 levels). It can be speculated that at the intrahepatic level, the anti-inflammatory Th2 pathway is stimulated as countermeasure to inhibit the systemic and hepatic Th1 up-regulation. Interestingly the Th2 response was common to DM and NASH. It has been demonstrated that IL4 was able to improve insulin sensitivity and glucose tolerance in an animal model of diet induced obesity [53], therefore its up-regulation could be a tentative to restore insulin sensitivity, which is a common pathogenetic mechanism in DM and NASH. A recent study has described an imbalance of the Th1/Th2 immune response towards an enhanced Th1 and a suppressed Th2 response in patients with DM and cardiovascular disease [40]. Since DM, NASH and cardiovascular disease are tightly correlated, it can be speculated that in NASH/DM co-morbidity the Th2 system (with a possible important role of the intrahepatic Th2 response) still attempts to counterbalance the Th1 up-regulation, while in the case of DM/cardiovascular co-morbidities the Th1 enhancement is predominant.

Liver biochemistry was further analyzed. In particular, higher transaminases correlated with a, possibly compensatory, anti-inflammatory response as shown by the negative correlation with the P-Th1/Th2 ratio and with the enhanced IL10 in comparison with the IL17. The latter phenomen was liver-specific in patients with NASH in comparison with patients without NASH, where it was a peripheral phenomeneon.

Taken together these data show that a pro-inflammatory cytokine state is associated with more disturbed histological, haemodynamic and metabolic features in NASH patients. An increase of the M1/M2 ratio and a decrease of the IL10/IL17 ratio play a key role in this process.
